# Genetic diagnosis of CYP21A2-related CAH: adaptive sampling long-read sequencing is an accurate and scalable solution

**DOI:** 10.1038/s41431-026-02019-8

**Published:** 2026-01-22

**Authors:** Dorte Launholt Lildballe, Morten Reiffenstein Huno, Lukas Ochsner Reynaud Ridder, Camilla Mains Balle, Simon Opstrup Drue, Agnethe Berglund, Morten Dunø, Ebbe Norskov Bak, Mette Hansen Viuff, Laura Skak Rasmussen, Claus Højbjerg Gravholt

**Affiliations:** 1 Department of Molecular Medicine, Aarhus University Hospital, Aarhus, Denmark; 2 Department of Clinical Medicine, Health, Aarhus University, Aarhus, Denmark; 3 Department of Endocrinology, Aarhus University Hospital, Aarhus, Denmark; 4 Department of Clinical Genetics, Aarhus University Hospital, Aarhus, Denmark; 5 Department of Clinical Genetics, Rigshospitalet, Copenhagen, Denmark; 6 Department of Gynaecology and Obstetrics, Aarhus University Hospital, Aarhus, Denmark; 7 Present Address: Department of Orthopedic Surgery, Slagelse Hospital, Slagelse, Denmark

**Keywords:** Genome assembly algorithms, Adrenal gland diseases

## Abstract

Congenital adrenal hyperplasia (CAH) is an autosomal recessive disorder, commonly caused by variants in *CYP21A2* (chr6p21.33), which encodes the 21-hydroxylase enzyme. Genetic diagnosis is challenging due to the high homology between *CYP21A2* and its nearby pseudogene *CYP21A1P*. The current gold standard, PCR-based Sanger sequencing combined with multiplex ligation-dependent probe amplification (MLPA), is labor-intensive, costly, and amenable to PCR bias. Furthermore, it is not reliable in detecting complex structural variants, and it provides no information on whether variants are located on the same allele or not. The purpose of this study was to develop a method based on long-read sequencing (LRS) for accurate diagnostics of *CYP21A2* variants and their phasing. Adaptive sampling (AS-)-LRS with chromosome 6 as region-of-interest was applied to DNA from 34 patients clinically diagnosed with CAH. To overcome mapping challenges in the highly homologous regions, we developed NanoCAH, a custom bioinformatic tool that accurately distinguishes between *CYP21A2* and *CYP21A1P* reads. Using AS-LRS and NanoCAH, we genetically confirmed *CYP21A2*-associated CAH in 32 (94%) of the patients, including reliable phasing of the variants without the need for parental testing. AS-LRS clarified previously ambiguous findings, including the detection of chimeric genes, deletions, and missed variants. Compared to current gold standard methods, AS-LRS proved to be faster and more scalable, while providing greater accuracy in detecting variants within the *CYP21A2* region. This makes AS-LRS a promising tool not only for CAH diagnosis but also for genetic testing in other regions with complex genomic architecture.

## Introduction

Congenital adrenal hyperplasia (CAH) comprises a group of autosomal recessive disorders with enzyme deficiencies at various steps in the adrenal steroidogenic cascade. In a Danish population study, the prevalence of CAH was estimated to 15:100,000 newborn females and 9:100,000 males [1]. In the vast majority (95%) of patients, CAH is caused by biallelic variants in the *CYP21A2* gene that encodes the 21-hydroxylase enzyme [2]. 21-hydroxylase is critical for the biosynthesis of cortisol and aldosterone [2, 3]. When lacking, precursors of cortisol and aldosterone are converted to androgens, resulting in androgen excess. From here on, we use the term 21OHD-CAH when referring to CAH due to 21-hydroxylase deficiency. Clinically, 21OHD-CAH manifests in two main forms: the classic type, which is further subdivided into salt-wasting and simple-virilizing forms, and the non-classic form, which is the least severe form. As most patients are compound heterozygous, disease severity is determined by the allele carrying the variant causing the least reduction in 21-hydroxylase activity [4].

*CYP21A2* is located in the so-called major histocompatibility complex (MHC) class III region of chromosome 6, alongside its inactive pseudogene *CYP21A1P* [5, 6]. The two genes are part of the RCCX module, which includes *RP*, *CYP21*, *C4*, and *TNX* [5, 6]. Due to the high sequence homology between *CYP21A2* and *CYP21A1P*—98% in the coding and 96% in the non-coding regions—combined with their arrangement within the RCCX module, unequal cross-over during meiosis is frequently seen. Most pathogenic alleles associated with 21OHD-CAH result from these recombination events, with gene conversions and asymmetric recombinations responsible for approximately 70% and 25% of cases, respectively [6, 7]. Briefly, gene conversions result in small conversions, where a single variant from the pseudogene is transferred to the gene, or in larger conversions, where a more extensive part of the pseudogene replaces part of the gene. Asymmetric recombination events create larger deletions, duplications or often lead to the formation of a non-functional chimeric gene with the 5’ end containing *CYP21A1P* and the 3’ end containing *CYP21A2* [6, 7]. Chimer*ic* genes are also seen in alleles carrying large gene conversions. Due to the high sequence homology, these events are difficult to distinguish in the *CYP21A* gene region. The current gold standard for genetic characterization of 21OHD-CAH is based on lPCR and Sanger sequencing combined with multiplex ligation-dependent probe amplification (MLPA) [8]. However, the combination of Sanger sequencing and MLPA is labor-intensive and expensive. In addition, this analytical strategy has several limitations. These include the ability to detect certain variants and structural alterations, such as a deletion on one allele being masked by a duplication of *CYP21A2* on the other allele, limited capacity to determine cis/trans position, and the risk of erroneously assigning disease-causing variants to the functional gene, when they in fact reside in the pseudogene [6, 8]. Short-read NGS cannot be used routinely, because the individual reads cannot be uniquely mapped to either *CYP21A2* or the pseudogene *CYP21A1P*.

This study evaluates the usefulness of adaptive sampling long-read sequencing (AS-LRS) for the genetic characterization of 21OHD-CAH, assessing its potential to overcome limitations of the current standard method and offering a more efficient, cost-effective, and precise approach for detecting complex variants in *CYP21A2*.

## Subjects and methods

### Subjects

The 34 patients for this study were part of a larger study on CAH, which was recently published [9]. Before inclusion in this study, a subpopulation of 24 patients (test samples) had previously undergone genetic testing using either hotspot testing (*N* = 12), long-range PCR based-Sanger sequencing (*N* = 2), long-range PCR based-Sanger sequencing and MLPA (*N* = 16) or method was not reported (*N* = 4) before being subjected to AS-LRSThe remaining 10 patients (validation samples) were tested using the conventional methods (long-range PCR based-Sanger sequencing and MLPA) after AS-LRS analysis. In all cases, the second method was performed blinded to the results of the initially applied method. All participants received oral and written information about the study before written consent was obtained. The study was approved by the local ethics committee (Central Region Denmark, Denmark (1-16-02-307-14).

### Sanger sequencing and MLPA

Sanger sequencing and MLPA were performed at The Molecular Genetic Laboratory, Department of Clinical Genetics (Rigshospitalet, Copenhagen, Denmark). PCR was performed with subsequent Sanger sequencing of the coding and exon-intron boundary regions of *CYP21A2* based on the method described previously [10]. Briefly, Sanger sequencing is based on the amplification of two PCR products spanning the entire *CYP21A2* locus (exonic and intronic regions), except for a 33 bp *CYP21A2-*specific sequence. This is followed by 12 Sanger sequencing reactions (forward and reverse). Data analysis was performed in Mutation Surveyor (SoftGenetics, LLC., PA, USA). If Sanger sequencing did not result in genetic confirmation of the CAH diagnosis, additional MLPA analysis was performed, using the SALSA MLPA kit P050B CAH (MRC Holland, The Netherlands) according to the manufacturer’s instructions.

### Adaptive sampling long-read sequencing (AS-LRS)

The AS-LRS was performed at the Department of Molecular Medicine (Aarhus University Hospital and Aarhus University, Aarhus, Denmark). DNA extraction was carried out using the QIAsymphony DNA Midi Kit as described by the manufacturer (Qiagen, Hilden, Germany). The DNA concentration of the extracts was quantified with the Qubit dsDNA Assay Kit; Life Technologies.

Compared to recommendations of the supplier (Oxford Nanopore Technology), sequencing libraries were prepared with an adjusted protocol using an input of 2–3 µg of DNA, RNase treatment (RNase Cocktail Enzyme Mix, Invitrogen), G-tube fragmentation (2500 x *g*) and library preparation with the Nanopore Ligation Sequencing Kit V14. The loading amount was 320–530 ng of library to FLO-PRO114M flowcells on PromethION 24 using MinKNOW version 24.06.10, and adaptive sampling was using full T2T-CHM13v2.0 as a reference and a bed-file with Chromosome 6 as the Region of Interest (ROI). In AS-LRS, ROI can be adjusted for each sample and hence also includes other genes/regions of interest, enriching for 1–10% of the human genome (according to the manufacturer). The combined pod5-files were basecalled with 5mC/5hmC detection at CpG-sites (v1) using the SUP model (v5.0.0) and dorado (v0.7.2). All data were processed and stored at GenomeDK (Aarhus University, Aarhus, Denmark). A summary of sequencing statistics is given in Supplementary Table S1. Sequencing data was aligned to the reference genome GRCh38 using minimap2 [11].

Since the ROI contains highly homologous regions, we designed the tool called NanoCAH (https://github.com/LauraSkak/NanoCAH). NanoCAH improves mapping and visualization in the range chr6:31900000-32200000 of GRCh38 to be able to identify the different alleles as accurately as possible. In short, the algorithm utilizes that reads are often mapped to both the active gene and the pseudogene. This makes it possible to find pairs of read groups, which can be used to choose the correct alignment of multi-mapped reads and identify large structural variants (SV). Single-nucleotide variants (SNVs) and small indels (<50 bp) were called based on the NanoCAH-filtered alignments using Clair3 [12]. SV were called based on the haplotype assemblies produced by NanoCAH using SVIM-asm [13].

### Variant filtering, classification, and visualization

#### Filtering

We used VarSeq v2.6.1 (Golden Helix Inc., Bozeman, MT) filtering for rare (≤1%) SNVs and indels (≤1%) in the GnomAD database (v 2.1.0) in ROI (chr6(GRCh38):31,980,000-32,112,000). All SV overlapping the same region were included in the filtering. Allele frequencies in gnomAD are based on short-read sequencing. Generally, this is problematic for difficult genomic regions, as these are underrepresented and/or information might be error-prone due to inaccurate read mapping. In this method development study, this proved not to be an issue as we identified all variants detected by previous methods.

#### Variant classification

Identified variants were classified according to ACMG/AMP guidelines [14]. Only class 3–5 variants are reported.

All variants in this publication are submitted to ClinVar (submission SUB15691254; SUB15860489).

#### Visualization

Alignments were visualized in Integrative Genomic Viewer (IGV) [15]; a guide to interpretation of the SV is given in the GitHub repository (https://github.com/LauraSkak/NanoCAH).

## Results

A total of 34 patients, who all had a clinical diagnosis of CAH, were included. Information on sex, age at inclusion, and CAH-subtype was obtained at the time of inclusion in the initial study (Table 1) [9].

**Table 1:** Summary of the distribution of sex, age at genetic testing, and CAH-subtype for included patients.

	Male	Female
*N* (**%**)	11 (32)	23 (68)
Age (y), median (iqr)	30.6 (24.8; 37.9)	35.8 (26.6; 50.4)
CAH type, *N* (%)		
Salt wasting	5 (45)	11 (50)
Simple virilising	4 (36)	2 (9)
Non-classic	2 (18)	9 (41)

Iqr: interquartile range. Y: years.

**Fig. 1:**
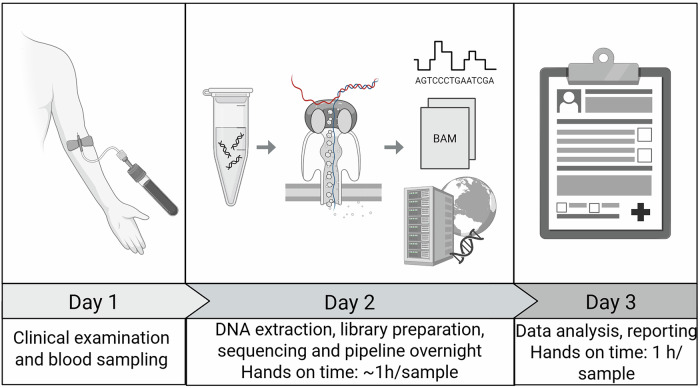
Graphical overview of the process from sample draw to clinical report using AS-LRS for genetic confirmation of CAH. The figure is made in BioRender (Created in BioRender. Gravholt, C. (2026) https://BioRender.com/ddvzox9). Hands-on time per sample depends on the number of samples being processed in parallel. A batch of 8 samples is processed in 6 h, resulting in an average hands-on time of less than 1 h per sample. Base calling runs in parallel with sequencing, and the analysis pipeline is automatically started once sequencing and base calling are complete. For comparison, Sanger sequencing and MLPA have a hands-on time of approximately 2.5 h/sample.

Our 2-day analytical setup is shown in Fig. 1. PCR-based LRS has all the disadvantages of Sanger sequencing, and hence we chose a PCR-free method: AS-LRS. In AS-LRS, only DNA fragments aligned to the chosen target (entire chromosome 6) were completely sequenced, optimizing the use of the on-target sequencing capacity of the instrument.

**Fig. 2:**
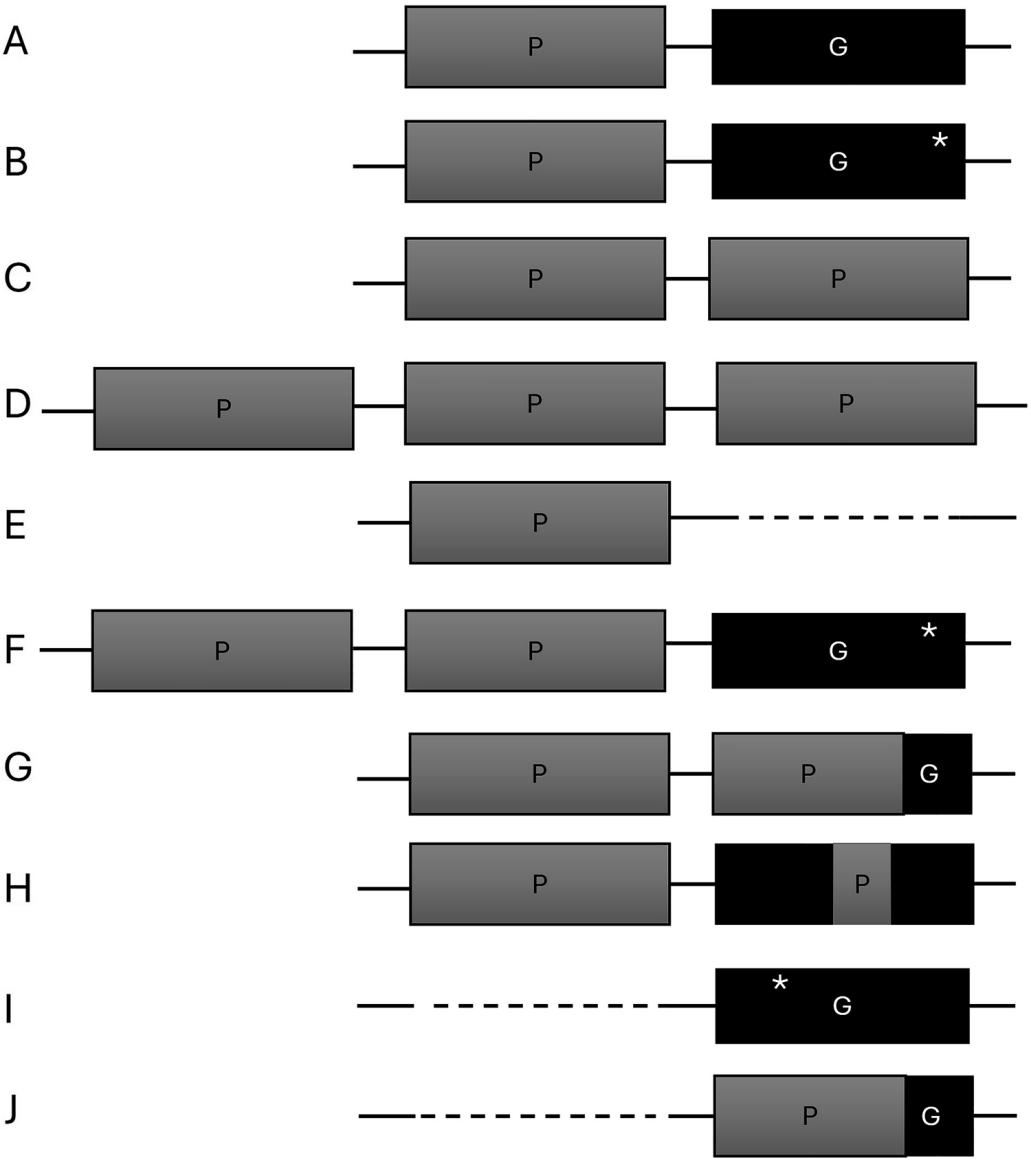
Graphic visualization of the different types of haplotypes identified. **A** wildtype configuration. **B** *CYP21A1P* intact, pathogenic variant in *CYP21A2*. **C** *CYP21A2* deleted, two copies of *CYP21A1P*. **D** *CYP21A2* deleted, three copies of *CYP21A1P.* **E** *CYP21A2* deleted, one copy of *CYP21A1P.* **F** two copies of *CYP21A1P* and pathogenic variant in *CYP21A2*. **G** Chimera where the majority of *CYP21A2* is replaced *by CYP21A1P*. **H** Chimera where part of *CYP21A2* is replaced *by CYP21A1P*. **I** *CYP21A1P* deleted, pathogenic variant in *CYP21A2*. **J** Allele where only a *CYP21A2/CYP21A1P* chimera is present (no intact pseudogene or gene).

The genetic findings for each patient are shown in Table 2, including results of phasing and genetic results according to gold standard methods. The overall type of variant is also indicated with reference to Fig. 2, and details are given in supplementary Fig. S1. For structural variants, variant interpretation is explained by several examples in our GitHub repository: https://github.com/LauraSkak/NanoCAH.

**Table 2:** Genetic finding in patients with 21OHD-CAH.

sample ID	Test or validation	Allele	AS-LRS genotype	Phases by AS-LRS^a^	Variant class ^b^	Diagnosis Confirmed	Difference to Previous test	Previous test method^e^	Allele type (Fig. 2)	allele no. ^d^
CAH01	Testing	1:	gene deletion	2	C5	yes	-	hotspot	J	19
		2:	gene/pseudogene chimera^f^		C5		-		H	8
CAH02	Testing	1:	gene deletion	2	C5	no; carrier	-	hotspot	E	18
		2:	-		-		-		A	0
CAH03	Testing	1:	gene deletion	2	C5	yes	-	hotspot	J	19
		2:	c.955C>T		C5		-		B	12
CAH04	Testing	1:	c.92C>T	2	C5	yes	-	hotspot	B	17
		2:	c.955C>T		C5		-		B	12
CAH05	Testing	1:	c.844G>T	2	C5	yes	-	hotspot	F	3I
		2:	c.955C>T		C5		-		B	12
CAH06	Testing	1:	c.293-13C>G	2	C5	yes	-	NR	B	16
		2:	gene deletion		C5		-		J	21
CAH07	Testing	1:	gene deletion	2	C5	yes	-	S + M	J	19
		2:	c.1451_1452delinsC		C5		-		B	9
CAH08	Testing	1:	gene deletion	2	C5	yes	-	hotspot	E	21
		2:	gene deletion		C5		-		J	18
CAH09	Testing	1:	gene deletion	2	C5	yes	-	S + M	J	19
		2:	c.1088C>T		C5		-		B	11
CAH10	Testing	1:	c.92C>T	2	C5	yes	-	S + M	B	17
		2:	gene deletion		C5		-		J	19
CAH11	Testing	1:	gene deletion	2	C5	yes	-	hotspot	D	1I
		2:	c.844G>T		C5		-		F	3I
CAH12	Testing	1:	gene deletion	1	C5	yes^c^	-	S + M	J	19
		2:	gene deletion		C5		-		J	19
CAH13	Testing	1:	c.293-13C>G	2	C5	yes	-	S + M	B	16
		2:	c.844G>T		C5		-		F	3I
CAH14	Testing	1:	c.518T>A	2	C5	yes	-	hotspot	B	15
		2:	c.518T>A		C5		-		B	15
CAH15	Testing	1:	c.293-13C>G	2	C5	yes	-	SS	B	16
		2:	gene/pseudogene chimera^f^		C5		-		H	7
CAH16	Testing	1:	gene deletion	2	C5	yes	-	NR	J	19
		2:	c.518T>A		C5		-		B	15
CAH17	Testing	1:	gene deletion	2	C5	yes	-	NR	J	19
		2:	c.518T>A		C5		-		B	15
CAH18	Validation	1:	c.293-13C>G	2	C5	yes	-	S + M	B	16
		2:	c.518T>A		C5		-		B	15
CAH19	Validation	1:	c.293-13C>G	1	C5	yes^c^	-	S + M	B	16
		2:	c.293-13C>G		C5		-		B	16
CAH20	Validation	1:	c.92C>T	2	C5	yes	-	S + M	B	17
		2:	c.518T>A		C5		-		B	15
CAH21	Validation	1:	c.293-13C>G	2	C5	yes	-	S + M	B	8
		2:	gene deletion		C5		-		I	22
CAH22	Validation	1:	gene deletion	2	C5	no, C5 and C3	-	S + M	J	21
		2:	c.1087G>A		C3		-		B	0
CAH23	Validation	1:	c.518T>A	2	C5	yes	-	S + M	B	15
		2:	c.923dup		C5		-		B	13
CAH24	Validation	1:	c.293-13C>G	2	C5	yes	-	S + M	B	16
		2:	c.293-13C>G		C5		-		B	16
CAH25	Validation	1:	c.92C>T	2	C5	yes	-	S + M	B	17
		2:	c.293-13C>G		C5		-		B	16
CAH26	Validation	1:	c.92C>T	2	C5	yes	-	S + M	B	17
		2:	c.293-13C>G		C5		-		B	16
CAH27	Validation	1:	c.92C>T	2	C5	yes	-	S + M	F	20
		2:	c.(518T>A;844G>T)		C5		-		F	2I
CAH28	Testing	1:	gene/pseudogene chimera^f^	2	C5	yes	p.Pro31Leu	hotspot	G	6
		2:	gene deletion		C5		p.Pro31Leu		J	19
CAH29	Testing	1:	c.293-13C>G	2	C5	yes	-	hotspot	B	16
		2:	gene deletion		C5		c.239-13C>G		J	19
CAH30	Testing	1:	c.188A>T	2	C5	yes	Novel in AS-LRS	hotspot	B	14
		2:	gene/pseudogene chimera^f^		C5		Deletion		G	5
CAH31	Testing	1:	c.518T>A	2	C5	yes	-	hotspot	B	15
		2:	c.1360C>T		C5		Novel in AS-LRS		B	10
CAH32	Testing	1:	gene/pseudogene chimera^f^	2	C5	yes	Deletion	S + M	G	4
		2:	c.844G>T		C5		-		F	3I
CAH33	Testing	1:	c.293-13C>G	2	C5	yes	-	NR	B	16
		2:	duplication of pseudogene, deletion of gene		C5		Deletion		C	4
CAH34	Testing	1:	c.293-13C>G	2	C5	yes	Uncertain	SS	B	16
		2:	gene/pseudogene chimera^f^		C5		Uncertain		H	7

^a^No of alleles possible to phase from AS-LRS data. 1 phase resembles identical alleles.

^b^ Variant classification is based on ACMG guideline [14].

^c^ homozygosity for the same pathogenic allele was detected. As AS-LRS does not have allele dropout, we consider these cases solved as well.

^d^ Numbers correspond to alleles presented graphically in supplementary Fig. S1.

^e^The previous test methods were either targeted testing for 8 common C4/C5 variants (hotspot), Sanger sequencing of the entire region (SS), or both Sanger sequencing and MLPA (S + M). In some of the early reports, the laboratory method was not reported (NR), and it was not possible to identify. Historically, the presence of an 8 bp deletion in the PCR product that should represent the gene was reported as 8 bp deletion in *CYP21A2*, although it may have been difficult to distinguish if this was due to chimeras or gene conversions.

^f^By Sanger sequencing of long-range PCR products, gene/pseudogene chimera may be detected as the presence of *CYP21A1P-like* variants in PCR fragments specific for the *CYP21A*2 instead of chimeras. The abnormal allele was detected, but the genetic background may be inaccurate. Hence, we use the term gene/pseudogene chimera for variant annotation.

In 30 of the 34 patient samples, we identified compound heterozygous causal variants in the *CYP21A2* gene/pseudogene regions (Table 2). In two patients (CAH12, CAH19), the alleles were identical, so phasing could not be confirmed, but since the method is without risk of PCR bias, and coverage was >30x, we consider these patients solved as well, resulting in a total diagnostic yield of 94%. In one patient (CAH22), we identified a pathogenic variant in trans with a variant of uncertain significance, and in one other patient (CAH02), we only detected one heterozygous pathogenic variant.

Compared to findings using the previous gold standard method, results were confirmed for most of the 68 alleles in our 34 patients. Patients CAH28-32 were previously tested by hot-spot testing. In two CAH30 and CAH31, a missense variant was not reported using the original methods. In two others (CAH28 and CAH29), the original reporting was a homozygous pathogenic SNV. AS-LRS identified the same SNV but in trans with a gene deletion. In other patients, AS-LRS clarified the nature of the underlying variant accurately; in patients CAH32 and CAH33, AS-LRS identified the causal allele as a chimera, whereas MLPA identified the allele as a deletion allele. In patient CAH34, multiple variants were previously reported, whereas with AS-LRS, we could phase the alleles and identify the causal variants as a single-SNV in one and a chimera in the other.

## Discussion

To our knowledge, this is the first study to utilize PCR-free AS-LRS for the genetic diagnosis of *CYP21A2*-associated CAH. Conventionally, the standard diagnostic method for CAH has relied on long-range PCR (800–2100 bp) combined with Sanger sequencing and MLPA [6]. Although these PCR-based methods have been key in identifying disease-causing variants, they have several limitations. Most prominent is PCR bias, which can lead to allelic dropout and misidentification of complex rearrangements [4, 6]. Also, in our study, 12 patients were originally only examined by hotspot testing (Table 2), which is also likely to miss rearrangements. In addition, PCR amplification of *CYP21A2* relies on unique sequence motifs for primer hybridization, where potential pathogenic variants will escape detection, as was the case for patient CAH34. In contrast, our PCR-free AS-LRS method inherently avoids these pitfalls, ensuring that both alleles are accurately represented during sequencing and that the entire *CYP21A2* sequence can be obtained.

Even when using AS-LRS, *CYP21A2* is difficult to resolve due to the homologous pseudogene *CYP21A1P*, located just upstream of *CYP21A2*. The proximity and high homology between the two render read mapping to the region ambiguous. High genomic instability in the area triggers large duplications and deletions and often results in chimeric genes. All these factors lead to inaccurate variant calls from this region. Even with the improved read lengths gained with LRS, this ambiguous read mapping still causes issues. NanoCAH is a software tool developed for this project to solve the problem of ambiguous read mapping. By employing this specialized bioinformatic algorithm, we can now accurately distinguish between reads mapping to the active *CYP21A2* gene and those mapping to the pseudogene. Furthermore, we can detect large deletions, insertions or chimeric genes, and phase reads much more reliably compared to using ordinary tools for structural variant calling and phasing with the original alignment.

More recently published methods using LRS for analysis of CAH have all relied on long-range PCR products as input material [16–18]. This may reduce cost and hands-on time, both compared to the golden standard and to AS-LRS. However, it does not remove the drawbacks of PCR-based methods, where uneven amplification of DNA may lead to spurious results. PCR-free LRS methods allow for accurate phasing of variants, which can be used to identify whether variants are in *cis* or *trans,* reducing the need for parental testing, thus saving both time and costs. In our study, AS-LRS clarified the phasing of variants in 94% of the patients, both patients with SNVs in different alleles and patients with SNV and SV in different alleles. In the remaining cases, phasing was not possible, most likely due to the presence of two identical alleles, as allele drop-out is unlikely in AS-LRS in contrast to PCR/Sanger sequencing.

Another issue associated with the phasing of variants is that long-range PCR is specifically challenging for alleles carrying multiple copies of *CYP21A2* in *cis*, which is not uncommon [8]. In itself, two copies on one allele are not pathogenic. But if using PCR-based methods (Sanger sequencing or LRS), there is a risk that two copies on one allele and zero copies on the other allele would be mistaken as one copy on each allele. This can result in erroneous test results both in diagnostic and carrier testing. If future treatments depend on knowing the true genetic background, this information is essential. Although none of our patients presented with such a finding, we included a validation sample with two copies on one allele. Using NanoCAH, we were able to detect this (See GitHub repository for examples).

Two patients remained without genetic confirmation of their clinical CAH diagnosis; one patient (CAH22) had a class 5 and a class 3 variant detected, and the other patient (CAH02) only had one variant detected [14]. In the first case, functional studies may be able to classify the C3 variant more accurately and clarify its potential pathogenic role. In the latter case, the disease could also be caused by variants in other genes associated with CAH (e.g., *CYP11B1, HSD3B2, CYP17A1, CYP11A1, POR*, and *STAR)*. These genes were not analyzed in this study, but since AS-LRS is flexible, these regions could easily be included in a future screening-based approach.

In the two patients (CAH30, CAH31) where a missense variant was not reported by tPCR/Sanger sequencing, the most likely explanation is that the previously unreported variants are located outside the eight common hotspot regions included in the original method. Prior to this study, patients CAH28 and CAH29 were also only tested by hot-spot Sanger sequencing. In one of them (CAH29), a gene deletion in *trans* with a pathogenic variant (SNV) was found using AS-LRS, where Sanger sequencing only detected the SNV as “homozygous”. Similarly, a homozygous missense variant was reported by Sanger sequencing, and by AS-LRS, we detected a gene deletion and a chimera (CAH28). It is possible that these variants would have been identified if Sanger sequencing and MLPA had been used instead of hot-spot testing. Still, these examples illustrate that AS-LRS can clarify the nature of the underlying variant more accurately, which is important when offering carrier testing to family members of variants in this complex genomic region, also securing accurate prenatal testing.

In three samples (CAH32, CAH33, CAH34), AS-LRS identified gene/pseudogene chimeras, which by MLPA are identified as an 8 bp deletion and hence *CYP21A1P*. The pathogenic allele was detected by the gold standard method, but its genomic nature was not necessarily accurate.

A different approach based on targeted capture using the PacBio-sequencing technology to carry out targeted-LRS has been described [19]. This approach overcomes the technical issues of PCR-based methods. On the downside, to get a reasonable cost balance, one needs to process a number of patient samples in parallel. This technology is therefore not practical for many clinical laboratories as either the turnaround time or the running costs would be too high [1]. In addition, each laboratory should consider costs for achieving the different LRS instruments and for implementing the bioinformatic pipelines needed to handle the data.

The recent, and likely continuous, decreasing costs for LRS in combination with full flexibility in scaling, in our setting, resulted in AS-LRS being compatible with previous methods. If only running one sample at a time, the hands-on times are comparable between PCR/MLPA-based methods and AS-LRS. One important advantage of our presented method is that sample preparation can be done in parallel for samples with different sequencing targets, reducing hands-on time to 30 min. Compared to MLPA, AS-LRS did not require any special method for DNA extraction or tissue type, although the latter was not explored in this study.

## Conclusion

In conclusion, compared to conventional methods, AS-LRS proved faster and more scalable, while providing greater accuracy in variant detection and haplotyping. Additionally, it provided information about phasing, reducing the need for parental testing. These features make AS-LRS a promising tool not only for CAH diagnosis, but also for genetic testing of other genomic regions with complex genomic architecture. This study may therefore pave the way for changing current golden standard method recommendations.

## Supplementary information

Supplementary Figure S1

Supplementary Table S1

## Data Availability

Restrictions apply to the availability of sequencing data generated and analyzed during this study to preserve patient confidentiality. The corresponding author will, on request, detail the restrictions and any conditions under which access to some data may be provided.

## Code availability

Bioinformatic tools developed for this study are available here: https://github.com/LauraSkak/NanoCAH.
