# Supplementary material for: Genetic diagnosis of CYP21A2-related CAH: adaptive sampling long-read sequencing is an accurate and scalable solution
Source: Eur J Hum Genet. 2026 Jan 22;34(4):535–42. doi: 10.1038/s41431-026-02019-8 (PMC13047035; doi:10.1038/s41431-026-02019-8)

**Supplementary Figure S1**

Figure S1: Illustration of the different alleles present among the 34 patients in the study. SNVs/indels are indicated by coloured dots in each allele for reads aligned to the *CYP21A2*. D: Deletion of CYP21A2 (G). I: insertion of at least one extra copy of *CYP21A1P*.

The allele numbers in this figure are also stated in Table 2 in the main manuscript to improve identification.

*SNVs/indel alelles*

In most of the analysed alleles, both *CYP21A1P* and *CYP21A2* were present and if a causal variant was detected, it was an SNV or indel in the gene. Alleles no 2-3, and 9-17 are of this type and is illustrated in Figure 2B in the main manuscript. In one case, *CYP21A1P* was deleted and one copy of *CYP21A2* was present in which a pathogenic SNV was detected, allele no 22 (Figure 2I).

*Deletion alleles*

Different types of deletion alleles were detected in this study where *CYP21A2* is deleted and either 1, 2, or 3 copies of *CYP21A1P* are present. This corresponds to allele numbers 1, and 18-21 and are illustrated in Figure 2C-D-E.

*Chimera alleles*

Chimeras are a mix of gene/pseudogene. In this study, we detected two major groups. In one group, *CYP21A1P* remains intact, and the 5’ end of *CYP21A2* is replaced with the corresponding 5’ sequence from *CYP21A1P* leaving a defect *CYP21A2* with only the 3’ sequence intact; this was identified in several different alleles – alleles no 4-6 (Figure 2G). In the other chimera group, *CYP21A1P* remains intact, and a central part of *CYP21A2* is replaced with the corresponding part of *CYP21A1P,* also leaving a defect *CYP21A2* with only the 5’ sequence intact. This is shown in allele no 7 and 8 (Figure 2H).


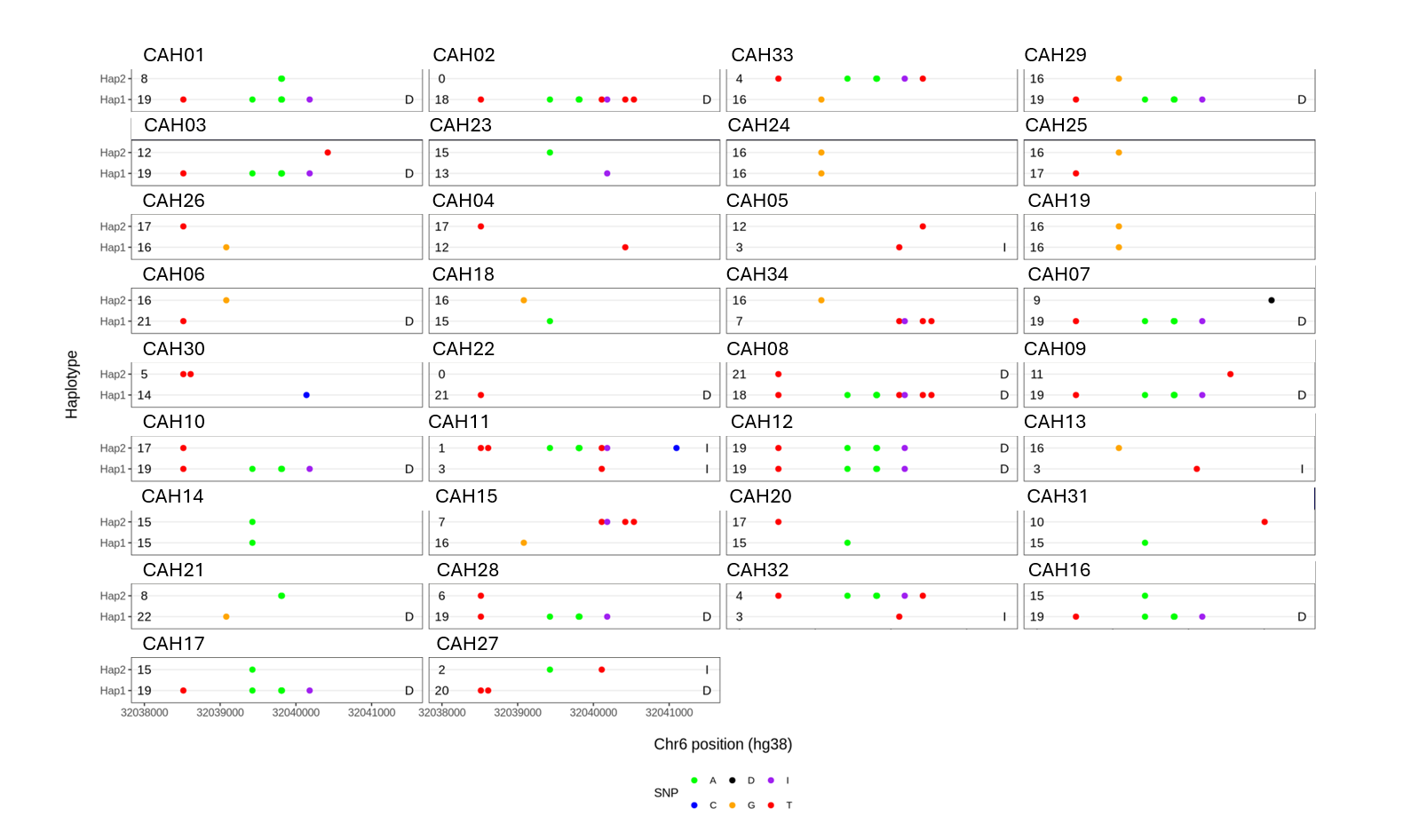

Supplement: Supplementary file 1 — Supplementary Figure S1 [file 41431_2026_2019_MOESM1_ESM.docx]
