# Supplementary material for: Genetic diagnosis of CYP21A2-related CAH: adaptive sampling long-read sequencing is an accurate and scalable solution
Source: Eur J Hum Genet. 2026 Jan 22;34(4):535–42. doi: 10.1038/s41431-026-02019-8 (PMC13047035; doi:10.1038/s41431-026-02019-8)
Supplement: Supplementary file 2 — Supplementary Table S1 [file 41431_2026_2019_MOESM2_ESM.docx]

Table S1: Median (range) values of selected quality parameters after AS-LRS of the included patient samples. Values reflect ROI¤

| Coverage | 41.5 (31-78) |
| --- | --- |
| Bases (GB) | 7.2 (5.4-13.4) |
| No or reads (mill) | 0.85 (0.66-1.7) |
| N50 (bp) | 11,220 (8,319-11,937) |
| Mean read length (bp) | 8,261 (6,219-9,333) |
| Longest read (kb) | 218,795 (107,424-779,037) |
| Median read length (bp) | 22.8 (20.1-25.6) |
| Mean read QUAL | 23.7 (20.9-26.7 |
| Median read QUAL | 24 (21-27) |
| Mean Block length (kb) | 179 (83-232) |
| Median block length (kb) | 52 (21-81) |
| N50 block length (kb) | 537 (355-676) |
| No blocks | 799 (628-1337) |
| Phased (%) | 83 (60-86) |

¤: ROI: region of interest; corresponds to chromosome 6 in this study
